# Sirtuin 1 regulates the phenotype and functions of dendritic cells through Ido1 pathway in obesity

**DOI:** 10.1038/s41419-024-07125-3

**Published:** 2024-10-18

**Authors:** Jean de Lima, Jefferson Antônio Leite, Paulo José Basso, Bruno Ghirotto, Eloisa Martins da Silva, Luisa Menezes-Silva, Meire Ioshie Hiyane, Carolina Purcell Goes, Luiz Lehmann Coutinho, Vinicius de Andrade Oliveira, Niels Olsen Saraiva Câmara

**Affiliations:** 1 Department of Immunology, Institute of Biomedical Sciences, University of São Paulo, São Paulo, Brazil; 2 Department of Animal Science, Luiz de Queiroz College of Agriculture (ESALQ), University of São Paulo, Piracicaba, Brazil; 3 Center for Natural and Human Sciences, Federal University of ABC, Santo André, Brazil

**Keywords:** Antigen-presenting cells, Dendritic cells

## Abstract

Sirtuin 1 (SIRT1) is a class III histone deacetylase (HDAC3) that plays a crucial role in regulating the activation and differentiation of dendritic cells (DCs) as well as controlling the polarization and activation of T cells. Obesity, a chronic inflammatory condition, is characterized by the activation of immune cells in various tissues. We hypothesized that SIRT1 might influence the phenotype and functions of DCs through the Ido1 pathway, ultimately leading to the polarization towards pro-inflammatory T cells in obesity. In our study, we observed that SIRT1 activity was reduced in bone marrow-derived DCs (BMDCs) from obese animals. These BMDCs exhibited elevated oxidative phosphorylation (OXPHOS) and increased extracellular acidification rates (ECAR), along with enhanced expression of class II MHC, CD86, and CD40, and elevated secretion of IL-12p40, while the production of TGF-β was reduced. The kynurenine pathway activity was decreased in BMDCs from obese animals, particularly under SIRT1 inhibition. SIRT1 positively regulated the expression of Ido1 in DCs in a PPARγ-dependent manner. To support these findings, ATAC-seq analysis revealed that BMDCs from obese mice had differentially regulated open chromatin regions compared to those from lean mice, with reduced chromatin accessibility at the Sirt1 genomic locus in BMDCs from obese WT mice. Gene Ontology (GO) enrichment analysis indicated that BMDCs from obese animals had disrupted metabolic pathways, including those related to GTPase activity and insulin response. Differential expression analysis showed reduced levels of Pparg and Sirt1 in BMDCs from obese mice, which was challenged and confirmed using BMDCs from mice with conditional knockout of *Sirt1* in dendritic cells (SIRT1∆). This study highlights that SIRT1 controls the metabolism and functions of DCs through modulation of the kynurenine pathway, with significant implications for obesity-related inflammation.

## Introduction

Sirtuins are a class of seven histones acetyltransferases (HATs) that can be found at the nucleus, cytosol, and/or mitochondria and are involved in the regulation of cell cycle, proliferation, differentiation and metabolism, and apoptosis of different types of cells [1–6]. Among the seven sirtuins, Sirtuin 1 (SIRT1) has been described as a chromatin modulator at regions of several histones [7] and can act on transcription factors important for the regulation of energy fitness, such as Peroxisome Proliferator-Activated Receptor Gamma Coactivator 1-Alpha (PGC-1α) and the Forkhead family of Transcription Factors 1 (FOXO1) and 3 (FOXO3) [8]. In addition, SIRT1 regulates the expression and function of proteins involved in cellular stress, DNA repair, and inflammation, highlighting its importance in the immune response and cellular metabolism [9–13].

Changes in the expression of SIRT1 were observed in several conditions including neurodegenerative diseases, cancer and aging [14]. The chronic low-grade inflammation steadily seen in obesity can impact cellular progenitors in bone marrow, including dendritic cells (DCs) [15, 16]. The deficiency of SIRT1 in DCs decreases the severity of arthritis by reducing costimulatory molecules, cytokine production, T cell proliferation and the Th1/Th17 effector response [17]. In obesity, SIRT1 is decreased in adipocytes [18, 19]. and in immune cells [20, 21]. Yet, the consequences of decreased SIRT1 in immune cells in obesity need clarification, especially the mechanisms involved in reprogramming DCs towards a pro-inflammatory phenotype, a hallmark of this condition.

Here, we report that SIRT1 expression was indeed downregulated in DCs in an experimental model of obesity. This reduction led to an increased DCs activation, and in turn Th1 phenotype. SIRT1 enhances PPAR-γ-mediated Indoleamine 2,3-Dioxygenase 1 (IDO1) regulation of tryptophan and kynurenine pathway, which seems disturbed in bone marrow-derived dendritic cells (BMDCs) from obese animals. The better understanding on how SIRT1 modulates the biology of DCs could provide new insights regarding treatments of metabolic inflammation.

## Results

### Obesity downregulates SIRT1 expression and activity in DCs

**Fig. 1:**
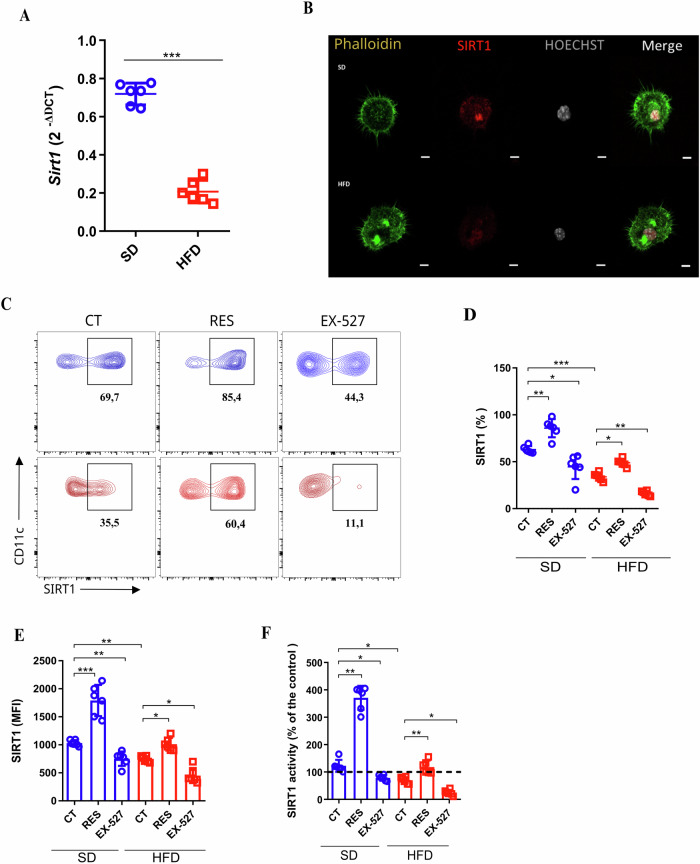
SIRT-1 expression is compromised in dendritic cells exposed to a high-fat diet. Bone marrow-derived dendritic cells (BM-DCs) were generated from mice under standard diet (SD) and high-fat diet (HFD). qPCR of Sirt1 from BMDCs from SD and HFD (**A**). Confocal microscopy of BMDCs/SD and BMDCs/HFD, showing SIRT1 (red) on BMDCs/SD and HFD (**B**). Flow cytometry representing the SIRT1 MFI (mean fluorescence intensity MFI) and percentage of SIRT1+ BMDCs (gated on Live & Dead-CD11c+IA/IE+CD115+) from SD and HFD animals, with or without RES (50 µM/24 h) and EX-527 (10 µM/24 h) (**C**–**E**). SIRT1 activity fluorometric assay of total protein extract from BMDCs from SD and HFD mice, formerly treated with RES (50 µM/24 h) or EX-527 (10 µM/24 h) or untreated (**F**). Significance values (*p*) are indicated as follows: *p < 0.05; **p < 0.01; ***p < 0.001; ****p < 0.0001, One-way ANOVA or t-test when necessary, using GraphPad Prism®. The graphs and illustrations represent a representative experiment of three distinct experiments with 3–6 animals per group.

Inflammation triggered by obesity downregulates the expression and activity of Sirtuin 1 (SIRT1) in adipocytes, monocytes [22] and neurons [23]. However, the impact of obesity on SIRT1 in dendritic cells (DCs) remains unknown. To address if SIRT1 is intrinsically impacted in DCs during obesity, we first demonstrated that the Sirt1 gene expression was downregulated in bone marrow-derived dendritic cells (BMDCs) from mice under a high-fat diet (HFD) (hereafter, BMDCs/HFD; Extended data Fig. 1a, b; Fig. 1A). Also, SIRT1 is located predominantly in the nuclei of BMDCs from lean mice (standard diet (SD)) (hereafter, BMDCs/SD; Fig. 1B). To complement our findings, we evaluated if the pharmacological activation of SIRT1 by resveratrol (RES) or specific inhibition by EX-527 (previously titrated by SIRT1 expression, Extended Fig. 1C–E) alters SIRT1 protein level and activity in DCs (Fig. 1C–E). The highest SIRT1 activity was observed in BMDCs/SD mice treated with RES. Conversely, EX-527 decreased SIRT1 activity in both BMDCs (Fig. 1F). Altogether, despite the decreased expression and activity of SIRT1 in BMDCs/HFD from mice, their levels and activity can be rescued or inhibited with pharmacological compounds.

### Reduced levels of SIRT1 in BMDCs/HFD induced pro-inflammatory profile and proliferation of IFN-γ-producing CD4 T cells

**Fig. 2:**
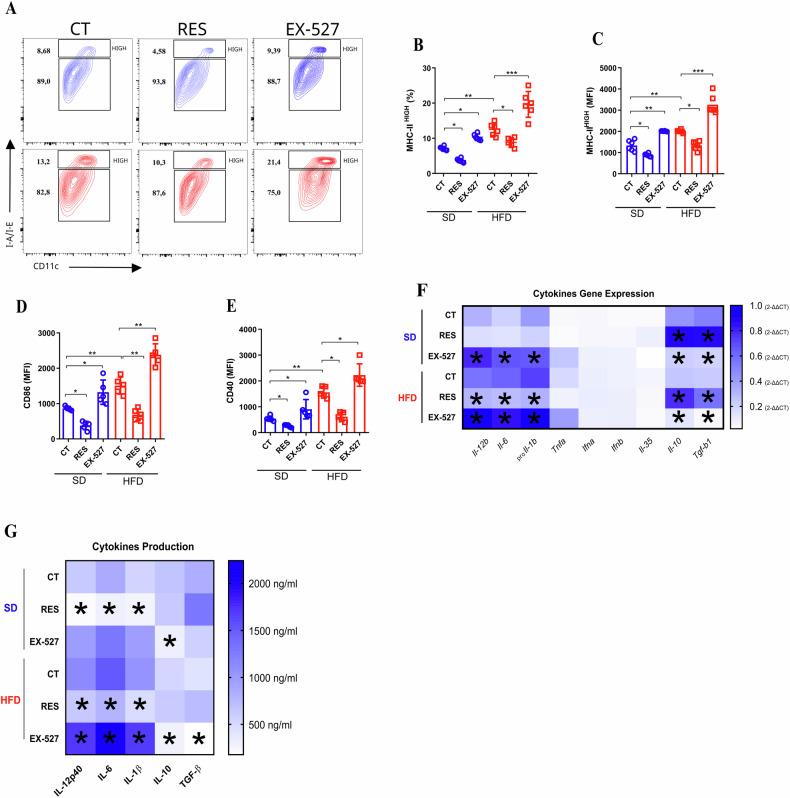
Pro-inflammatory activation of dendritic cells by a high-fat diet is inversely related to SIRT1 expression. Bone-marrow-derived Dendritic cells (BM-DCs) were generated from mice on a standard diet (SD) and a high-fat diet (HFD). MHC-II^high^ (top gate) and MHC-II^low^ (bottom gate) contour plot from flow cytometry of BMDCs from SD and BMDCs from HFD (with RES [50 µM], EX-527 [10 µM], or no treatment) (**A**). Quantification of percentage (**B**) and MFI (**C**) (mean fluorescent intensity) of MHC-II^high^ is represented. MFI (mean fluorescent intensity) of CD86 (**D**) and CD40 (**E**) from the same BMDCs under the mentioned conditions. Heat-map representing qPCR data (2-ΔΔCT) of the main cytokines produced by BMDCs/SD and BMDCs/HFD, untreated or treated with RES [50 µM] and EX-527 [20 µM] for 24 h (**F**). Heat-map of cytokine production was measured by ELISA for IL-1β, IL-6, IL-12p40, IL-10, and TGF-β in BMDCs from SD and HFD under the same conditions (**G**). The significance values (*p*) are indicated as follows: *p < 0.05; **p < 0.01; ***p < 0.001; ****p < 0.0001, determined by One-way ANOVA or t-test when necessary, using GraphPad Prism®. The graphs and illustrations represent a representative experiment of three distinct experiments, with 3–6 animals per group.

To evaluate the role of SIRT1 levels in BMDCs/HFD, we analyzed the expression of pro-inflammatory stimulatory molecules and cytokines expressed during dendritic cell-dependent immune synapsis. We initially observed that BMDCs/HFD increased Class II Major Histocompatibility Complex (MHC class II) and costimulatory molecules expression, such as Cluster of Differentiation 86 (CD86) and Cluster of Differentiation 40 (CD40) (Fig. 2A–E). Also, they had a different gene expression profile of cytokines with high expression of Interleukin-12 subunit p40 (IL-12p40), Interleukin-6 (IL-6), and Pro-Interleukin-1 beta (proIL-1β) (Fig. 2F; Extended data Fig. 2a–c). Conversely, BMDCs/SD had a higher expression of Interleukin-10 (IL-10) and Transforming Growth Factor Beta 1 (TGF-β1) (Extended data Fig. 2). RES treatment decreased costimulatory markers and IL-12p40 and IL-6 levels while also promoting IL-10 and TGF-β production in both BMDCs/SD and BMDCs/HFD (Fig. 2G). Blocking SIRT1 with EX-527 led to the opposite effect observed in both BMDCs (Fig. 2F, G). Thus, SIRT1 downregulated in BMDCs/HFD led to a pro-inflammatory profile, and the control of SIRT1 levels is directly related to BMDCs activation.

**Fig. 3:**
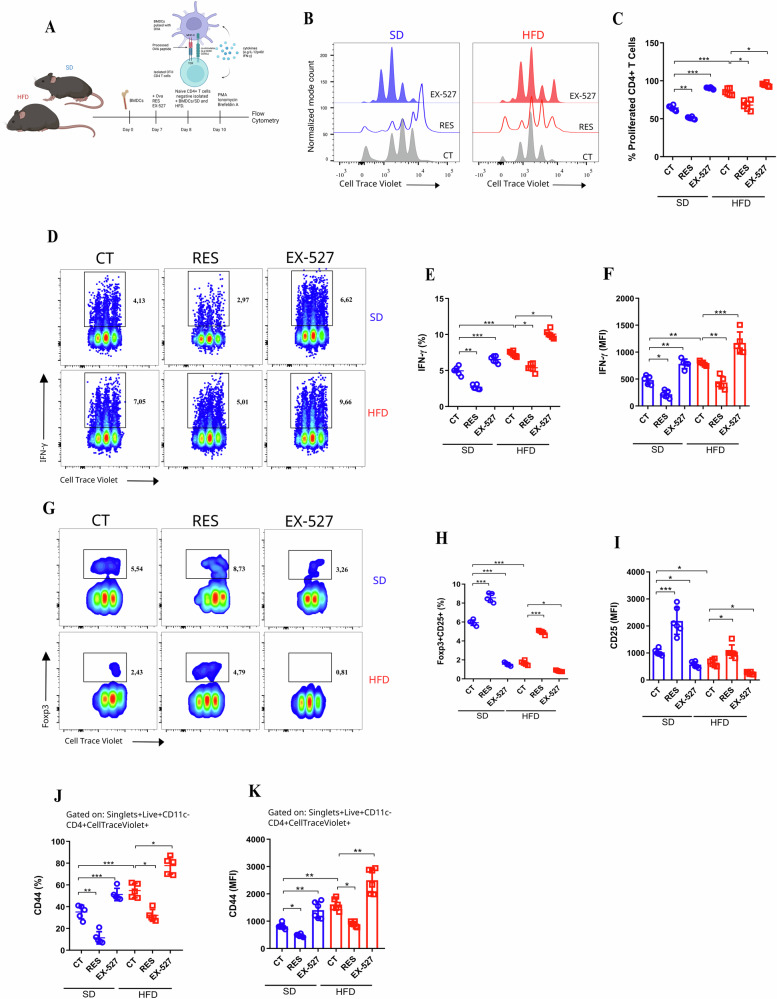
High-fat diet-induced pro-inflammatory DC favors Th1 generation by SIRT1 levels. Experimental design representation for BMDCs (OVA pulsed)-CD4 T cells (OT-II) co-culture approach (**A**). Histogram from flow cytometry representing Cell-Trace Violet-labeled OT-II CD4 T cells proliferation, which were co-cultured (72 h) with OVA-pulsed BMDCs/SD and BMDCs/HFD under RES [50 µM], EX-527 [10 µM] treatment, or untreated (CT) (**B**). Percentage of OT-II CD4 T cells proliferation (Cell-Trace Violet) after co-culture (72 h) with BMDCs/SD and BMDCs/HFD under treatment or no treatment (RES, EX-527, or CT respectively) (**C**). Pseudo color plot (**D**), percentage (**E**), and MFI mean fluorescence intensity) (**F**) of IFN-γ production from CD4 T cells (OT-II) co-cultured with BMDCs/SD and BMDCs/HFD under RES, EX-527 treatment, or no treatment (CT). Pseudo color smooth plot (**G**), percentage (**H**) of Foxp3 and CD25, and MFI (MFI mean fluorescence intensity) (**I**) of regulatory CD4 T cells (OT-II) previously co-cultured with BMDCs/SD and BMDCs/HFD under RES, EX-527 treatment, or no treatment. Percentage of CD44 (**J**) and MFI (mean fluorescence intensity) of CD44 (**K**) from CD4 T cells (OT-II) co-cultured with BMDCs/SD and BMDCs/HFD treated (RES or EX-527) or untreated. *p < 0.05; **p < 0.01; ***p < 0.001; ****p < 0.0001, determined by One-way ANOVA or t-test when necessary, using GraphPad Prism®. The graphs and illustrations represent a representative experiment of three distinct experiments, with 3–6 animals per group.

Next, we aimed to evaluate whether differential SIRT1 levels in BMDCs from standard diet (SD) and high-fat diet (HFD) mice would impact T cell activation, proliferation, and differentiation through an antigen-specific approach. To achieve this, we performed a co-culture of ovalbumin (OVA)-pulsed BMDCs and naive OT-II CD4 T cells. In this context, BMDCs were incubated with the antigen ovalbumin, which allows these cells to present the antigen to T cells (Fig. 3A; Extended data 3a and b). RES-treated BMDCs reduced the proliferation of antigen-specific CD4 T cells, while EX-527 enhanced it (Fig. 3B, C). Further, BMDCs/HFD induced higher expression of Interferon-gamma (IFN-γ) in CD4+ T cells, which was even increased by EX-527 (Fig. 3D–F). In contrast, BMDCs/SD induced a significant frequency of CD25+Foxp3+ in CD4 T cells (Fig. 3G, H; Extended data 3c), which was increased by RES in both BMDCs (Fig. 3H, I). Regarding the activation, BMDCs/HFD induced greater expression of CD44+ in CD4 T cells (Fig. 3J, K). As a side note, we did not observe Th2 or Th17 phenotypical changes when we used the same experimental approach (data not shown). Therefore, the reduced expression of SIRT1 in BMDCs/HFD led to enhanced activation, proliferation, and IFN-γ producer CD4 T cells in vitro.

### SIRT1 is a key regulator of canonical metabolic pathways in BMDCs

**Fig. 4:**
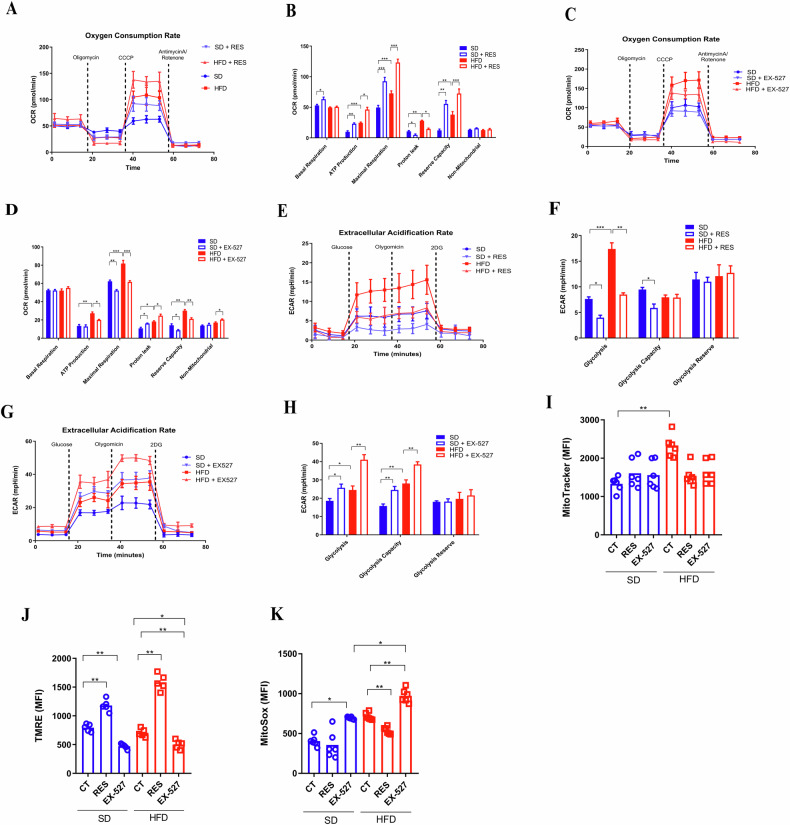
SIRT1 is a key regulator of canonical metabolic pathways in BMDCs. SIRT1 levels regulate metabolism of DCs. Oxygen consumption rate (OCR [pmol/min]) from Mitostress Assay of BMDCs/SD and BMDCs/HFD under RES [50 µM] OR no treatment (CT [DMSO]) (**A**, **B**). OCR of BMDCs/SD and BMDCs/HFD under EX-527 [10 µM] or CT (**C**, **D**). Extracellular Acidification rate (ECAR [mph/min]) of BMDCs/SD and BMDCs/HFD under RES [50 µM] or CT (**E**, **F**). ECAR of BMDCs/SD and BMDCs/HFD under EX-527 [10 µM] or CT (**G** and **H**). Mitotracker Green (MFI mean fluorescence intensity) (**I**), TMRE (MFI) (**J**) and MitoSox (MFI) (**K**) of BMDCs/SD and BMDCs/HFD under RES [50 µM], EX-527 [10 µM] or CT. The significance values (p) followed the order: *p < 0.05; **p < 0.01; ***p < 0.001; ****p < 0.0001, which were treated by the One-way ANOVA test or t-test when necessary, using GraphPad Prism®. The graphs illustrations are a representative experiment of three distinct experiments, which contained 3–6 animals per group.

Sirtuin 1 (SIRT1) acts by regulating different targets related to metabolism [23, 24], in caloric restriction and obesity animal models [25]. Additionally, the pro-inflammatory profile observed in our previous experiments has been correlated with both glycolysis [26, 27] and oxidative phosphorylation (OXPHOS) [27–29]. However, so far, these canonical metabolic pathways in dendritic cells (DCs) were not correlated with SIRT1 in obesity. To better understand this possible correlation, we performed an automatic measurement of energy metabolism in real time. The Oxygen Consumption Rate (OCR) in BMDCs/HFD was higher than in BMDCs/SD. In addition, resveratrol (RES) increased the OCR in both BMDCs/SD and BMDCs/HFD (Fig. 4A). ATP production, maximal respiration, and spare respiratory capacity were also enhanced. RES reduced proton leak in BMDCs/SD and, especially, in BMDCs/HFD (Fig. 4B). EX-527 decreased the OCR (Fig. 4C), ATP production, maximal respiration, and spare respiratory capacity in BMDCs in both conditions. Conversely, proton leak was increased after EX-527 treatment. BMDCs/HFD had a higher extracellular acidification rate (ECAR), while RES reduced it in both BMDCs, especially in BMDCs/SD (Fig. 4E, F). EX-527 treatment increased the ECAR in both BMDCs from SD and HFD mice, with higher levels in BMDCs/HFD (Fig. 4G, H).

We also evaluated the mitochondrial mass, membrane potential, and reactive oxygen species (ROS) production of those BMDCs/SD and BMDCs/HFD. Interestingly, RES and EX-527 had no effect on the mitochondrial mass (Fig. 4I). Nevertheless, RES treatment reduced the mitochondrial membrane potential and ROS production, while EX-527 boosted the mitochondrial membrane potential and ROS production (Fig. 4J, K). Together, these results indicate that obesity alters mitochondrial metabolism, suggesting a link between SIRT1 and metabolism in determining DCs activation fate.

### The kynurenine pathway regulates SIRT1 in DCs

Low SIRT1 levels in BMDCs/HFD affect both glycolysis and OXPHOS metabolic pathways, highlighting the complexity of SIRT1’s role in metabolism. Consequently, we opted to take a step back and investigate whether the decreased SIRT1 levels are primarily influenced by a reduced availability of essential metabolic coenzymes or if SIRT1 could regulate downstream targets responsible for providing functional coenzymes, thereby creating a positive feedback loop that enhances SIRT1 functionality.

**Fig. 5:**
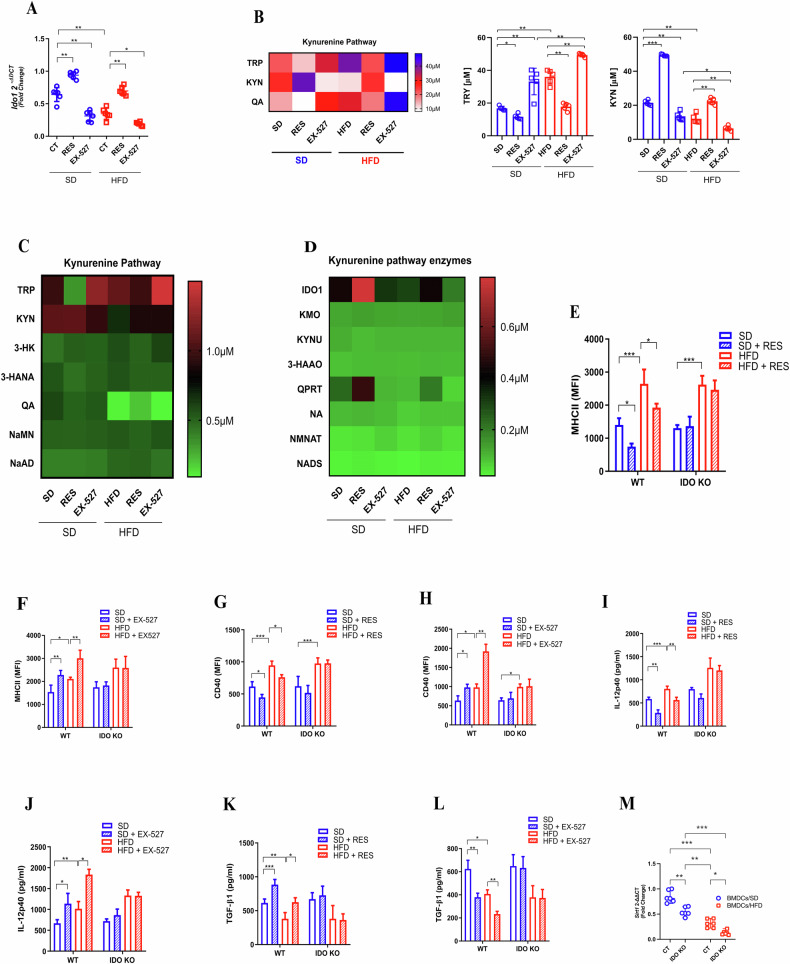
Decreased IDO1 in BMDCs induced by a high-fat diet can be modulated by SIRT1. qPCR of Ido1 from BMDCs/SD and BMDCs/HFD, under RES [50 μM], EX-527 [20 μM] treatment or no treatment (**A**). Liquid Chromatography coupled to Mass Spectrometry (LC–MS/MS) of kynurenine pathway intermediates molecules, Tryptophan (TRP), Kynurenine (KYN) and Quinolinic Acid (QA), from BMDCs/SD and BMDCs/HFD, under RES, EX-527 treatment or no treatment (**B**). LC–MS/MS Kynurenine pathway enzymes (Indoleamine dioxygenase 1(IDO1), Kynurenine 3-monooxygenase (KMO), Kynureninase (KYNU), 3-Hhydroxyanthranillic acid dioxygenase (3-HAAO), quinolinate phosporibosyltransferase (QPRT), Neuraminidase (NA), mononucleotide adenylyltransferase (NMNAT) and Nicotinamidase (NADS)) from BMDCs/SD and BMDCs/HFD, under RES, EX-527 treatment or no treatment (**C**). LC–MS/MS Kynurenine pathway metabolites (Tryptophan (TRP), Kynurenine (KYN), Quinolinic Acid (QA), 3-Hydroxykynurenine 3-HK), 3-hydroxyanthranillic acid (3-HANA), nicotinic acid mononucleotide (NaMN) and nicotinic acid mononucleotide (NaMN)) from BMDCs/SD and BMDCs/HFD, under RES, EX-527 treatment or no treatment (**D**). MFI of MHC-II, and CD40 from BMDCs/SD and BMDCs/HFD, under RES, EX-527 treatment or no treatment, extracted from WT and IDOKO (Ido Knockout) mice (**E**–**H**). ELISA of IL-12p40 and TGF-β (pg/ml) in the supernatant from BMDCs/SD and BMDCs/HFD, under RES, EX-527 treatment or no treatment, extracted from WT and IDOKO mice (**I**–**L**). qPCR analysis of Sirt1 in BMDCs from SD and HFD groups, including BMDCs from WT and IDO KO mice (**M**). The significance values (p) followed the order: *p < 0.05; **p < 0.01; ***p < 0.001; ****p < 0.0001, which were treated by the One-way ANOVA test or t-test when necessary, using GraphPad Prism®. The graphs illustrations are a representative experiment of three distinct experiments, which contained 3–5 animals per group.

Previous studies have identified Indoleamine 2,3-Dioxygenase 1 (IDO1) as one of the principal regulatory molecules in the biology of DCs, closely associated with the Nicotinamide Adenine Dinucleotide (NAD+) de novo pathway [30]. SIRT1 activity is influenced by the availability of essential metabolic coenzymes, such as NAD+, which acts as a cofactor for SIRT1-mediated deacetylation reactions [31] This suggests the existence of a potential feedback mechanism that amplifies SIRT1 functionality. Also, as with SIRT1, we observed that BMDCs/HFD had lower expression of IDO1 and RES restored IDO1 expression while EX-527 reduced it (Fig. 5A).

IDO1 converts tryptophan (TRP) into kynurenine (KYN), which can either be secreted into the microenvironment or subsequently cleaved by different enzymes until NAD+ generation [32]. The supernatant from BMDCs/SD culture had more KYN and decreased TRP, combined with the modulation of these molecules by RES and EX-527 (Fig. 5B, C). Regarding the enzymes of the KYN pathway, only IDO1 and Quinolinic Acid Phosphoribosyltransferase (QRPT) were negatively affected in BMDCs/HFD and with decreased function of SIRT1 by EX-527(Fig. 5D).

Next, we decided to check if IDO1 was responsible for the phenotypes observed in BMDCs with reduced SIRT1 levels, such as in BMDCs/HFD. In BMDCs/SD and BMDCs/HFD generated from IDO1 knockout mice (IDO KO), RES and EX-527 had no effect on MHC class II, CD40, IL-12p40, and TGF-β expressions (Fig. 5E–L), which were previously observed in BMDCs generated from wild-type mice (WT). Additionally, Sirt1 expression was reduced in BMDCs from IDO KO mice, suggesting again a loop effect between IDO1 and SIRT1 (Fig. 5M). In summary, these data shows that the effects of SIRT1 levels on BMDC phenotype correlated with IDO1 and an active KYN pathway, which are both affected in obesity.

### The impaired kynurenine pathway in BMDCs/HFD led to lower levels of NAD^+^ and reduced SIRT1 functionality

**Fig. 6:**
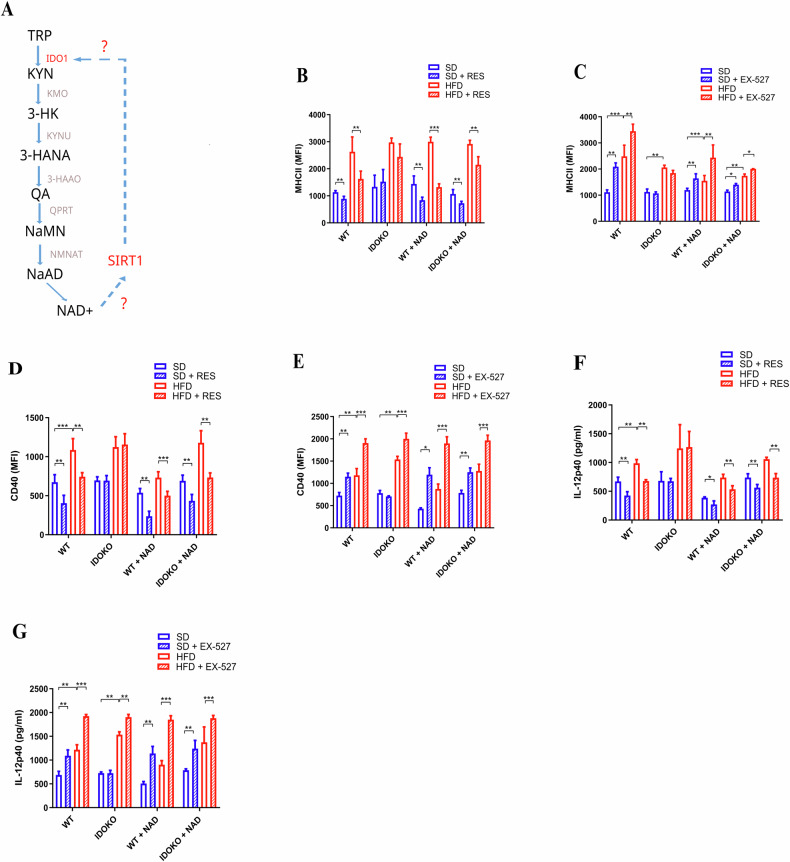
IDO1 can support SIRT1 functionality in BMDCs through NAD+ synthesis. Kynurenine pathway schema representing the hypothesis of the IDO1-SIRT1 axis (**A**). MFI (mean fluorescent intensity) of MHC-II and CD40 of BMDCs (with or without 2 mM of Acetylated-NAD) extracted from WT or IDOKO mice on 12 weeks of SD or HFD treatment (**B**–**E**). ELISA of IL-12p40 in BMDCs supernatant (with or without 2 mM of Acetylated-NAD) extracted from WT or IDOKO mice on 12 weeks of SD or HFD treatment (**F** and **G**). The significance values (p) followed the order: *p < 0.05; **p < 0.01; ***p < 0.001; ****p < 0.0001, which were determined by the One-way ANOVA test or *t*-test when necessary, using GraphPad Prism®. The graphs and illustrations represent a representative experiment of three distinct experiments, with 3–6 animals per group.

The intracellular sources of Nicotinamide Adenine Dinucleotide (NAD+) are diverse, and one of the main sources is the tryptophan-kynurenine pathway [33, 34]. We investigated NAD+‘s role on Sirtuin 1 (SIRT1) activity and, consequently, in BMDCs phenotypes (Fig. 6A). We opted to add acetylated-NAD in BMDCs culture to see whether it could rescue the effects of resveratrol (RES) and EX-527 in samples from IDO1 knockout mice (IDO KO) under standard diet (SD) and high-fat diet (HFD). We observed that acetylated-NAD treatment rescued the reduction of MHC class II mean fluorescence intensity (MFI), which was not observed in BMDCs derived from IDO KO mice (Fig. 6B, C). The same effect was rescued in BMDCs from IDO KO regarding CD40 and IL-12p40 levels (Fig. 6D–G).

Additionally, we sought to understand whether obesity or RES treatment could impact IDO1 expression in humans. Analysis of the biopsies of white adipose tissue from obese patients treated with 150 mg/day RES for 30 days revealed an increase in IDO1 expression compared with obese patients in the placebo group (Extended data 4a). In another study, we investigated IDO1 expression in human white adipose tissue samples from obese subjects obtained before (*n* = 16) and after bariatric surgery (*n* = 16) [35]. Again, IDO1 expression was increased after the bariatric surgery (BA) (Extended data 4b). SIRT1 levels were increased in lean patients compared with the obese group, which was restored after BA (Extended data 4c).

Together, IDO1 activity-induced NAD+ is an essential pathway to sustain SIRT1 expression, which regulates the phenotype of BMDCs/SD and BMDCs/HFD. This pathway might be conserved in humans.

### Obesity inherently affects SIRT1 levels at the epigenetic level in dendritic cells

**Fig. 7:**
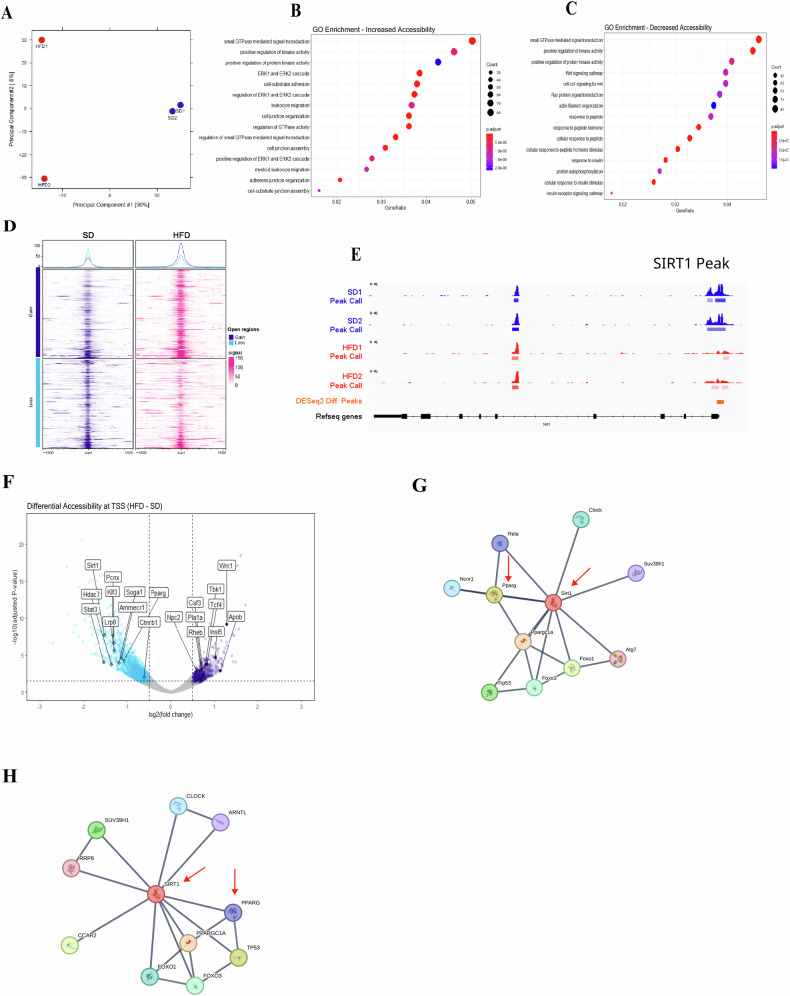
Obesity inherently affects SIRT1 levels at the epigenetic level in dendritic cells. Principal Component Analysis (PCA) from ATAC-seq of BMDCs/SD and HFD replicates (**A**). GO Enrichment analysis from increased and decreased accessibility at promoters of targets in different pathways (*p* < 0.01) (**B**, **C**). Global chromatin accessibility analysis from ATAC-seq of BMDCs/SD and HFD at promoters (**D**). Genomic SIRT1 locus with accessibility and differential accessibility peaks from ATAC-seq of BMDCs/SD and HFD (**E**). Volcano plot of differential accessibility in promoter regions from BMDCs/SD and HFD ATAC-seq data (**F**). Protein-Protein Interaction Networks Functional Enrichment Analysis (STRING) revealing high scores of confidence (>0.900) for PPARγ and interaction with SIRT1 in mice (**G**) and human (**H**). The significance values (*p*) or adjusted *p*-values followed the order: *p < 0.05; **p < 0.01; ***p < 0.001; ****p < 0.0001, which were determined by ANOVA test or t-test when necessary, using GraphPad Prism®. The graphs and illustrations represent a representative experiment from one to three distinct experiments, with 2–6 animals per group.

Finally, we investigated if the downregulation of Sirtuin 1 (SIRT1) in dendritic cells (DCs) derived from high-fat diet (HFD) mice would directly affect the chromatin conformation state and, consequently, IDO1 expression. Thus, we performed ATAC-seq on BMDCs/SD and BMDCs/HFD. Principal Component Analysis (PCA) reveals distinguished chromatin organization between BMDCs from lean and obese mice (Fig. 7A). Principal component 1 explains 90% of the difference between SD and HFD, while principal component 2 reflects the variation observed in the HFD samples. The assay revealed a total of 91,167 differential accessible peaks; of these, 7668 gained significant accessibility and 5202 were lost in HFD compared to SD (Fig. 7B).

Gene ontology (GO) enrichment analysis revealed increased accessibility in BMDCs/HFD chromatin for pathways regulated by GTPase and Extracellular Signal-Regulated Kinase 1/2 (ERK1/2) (Fig. 7C), pivotal signaling proteins for activating diverse immune functions [36, 37]. These proteins orchestrate cell structure, directionality, and the movement and proliferation of immune cells. Conversely, the Wnt signaling pathway and insulin-responsive pathways exhibited decreased accessibility in BMDCs/HFD. These findings suggest that BMDCs imprint a pro-inflammatory profile, potentially contributing to chronic immune responses in obesity, which is combined with lower accessibility of the Sirt1 genomic region in those cells (Fig. 7E).

Next, we sought the differential accessibility of transcription start sites (TSS) of genes that could be responsible for regulating IDO1. Among the candidates, Peroxisome Proliferator-Activated Receptor Gamma (PPARγ) in BMDCs/HFD (Fig. 7F). This has been described as a regulator of the kynurenine pathway and IDO1 in macrophages and DCs [38, 39]. Protein–protein Interaction Networks Functional Enrichment Analysis (STRING) also revealed strong evidence (with data published support) of high scores of confidence (>0.900) for PPARγ and interaction with SIRT1 in mice (Fig. 7G) and human (Fig. 7H).

In conclusion, our findings indicate that the diminished SIRT1 levels observed in DCs from obese mice result in reduced accessibility at the transcription start sites (TSS) of PPARγ. This alteration likely contributes to the downregulation of IDO1 expression and activity, presumably mediated by SIRT1 (Fig. 7F).

### Sirt1 conditional knockout in dendritic cells affects the global in vivo metabolic profile

**Fig. 8:**
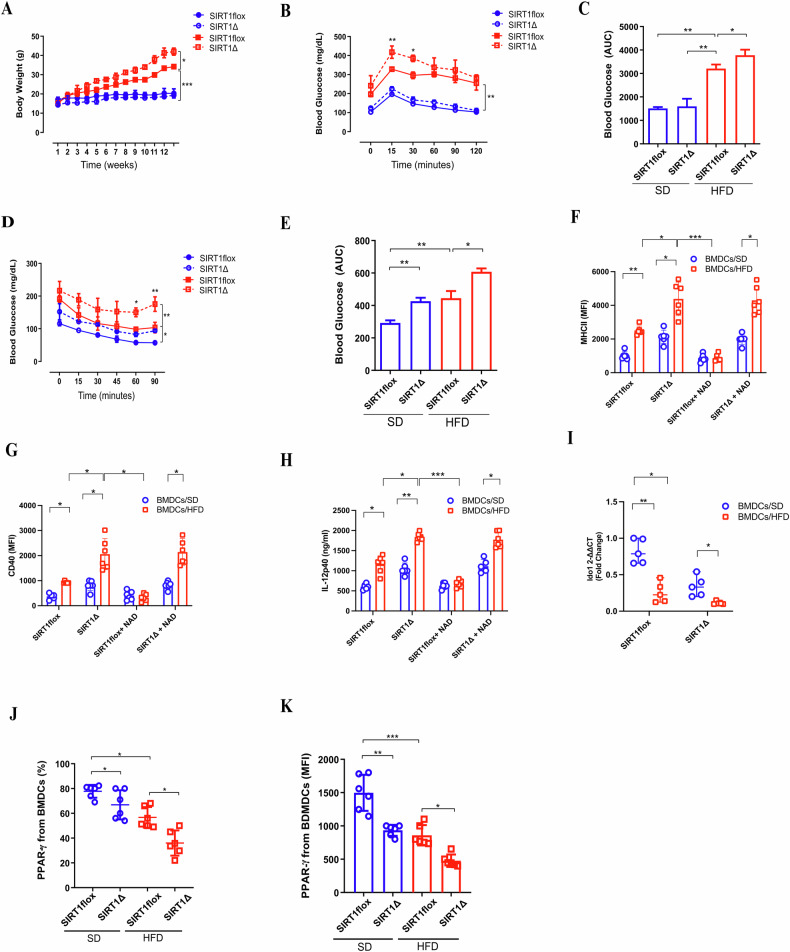
Conditional knockout of Sirt1 in dendritic cells disrupts global metabolism, possibly affecting NAD+ production via the kynurenine pathway. Body weight (g) from SIRT1Δ and control mice (SIRT1 flox) on a standard diet (SD) and high-fat diet (HFD) (**A**). Blood glucose tolerance test (GTT) levels, including Area Under the Curve (AUC) data (**B**, **C**). Insulin resistance test with AUC (**D**, **E**). Mean Fluorescent Intensity (MFI) of MHC-II, CD40, and ELISA of IL-12p40 in BMDCs (with or without 2 mM of Acetylated-NAD) extracted from SIRT1Δ and SIRT1 flox mice after 12 weeks on SD or HFD (**F**–**H**). Ido1 qPCR in BMDCs from SIRT1Δ and SIRT1 flox mice on SD and HFD (with or without 2 mM of Acetylated-NAD) (**I**). Intracellular flow cytometry staining of Peroxisome Proliferator-Activated Receptor Gamma (PPARγ) for percentage (**J**) and MFI (**K**) in BMDCs from SIRT1Δ and SIRT1 flox mice on SD and HFD. Significance values are indicated as follows: *p < 0.05; **p < 0.01; ***p < 0.001; ****p < 0.0001. Statistical analyses were performed using ANOVA or t-test where appropriate, with data analyzed using GraphPad Prism®. The graphs and illustrations represent data from one to three independent experiments, with 2–6 animals per group.

Resveratrol and EX-527 are reliable pharmacological tools for investigating SIRT1 functions in various cells and contexts. To refine our findings, we generated a Sirt1 conditional knockout mouse strain (SIRT1∆) to specifically assess whether the absence of SIRT1 in dendritic cells affects the pro-inflammatory and metabolic profiles associated with obesity in vivo. We observed that SIRT1∆ mice gained more weight on a HFD (Fig. 8A), exhibited lower glucose tolerance, and had higher insulin resistance (Fig. 8B–E). Interestingly, BMDCs/SD from SIRT1∆ mice displayed elevated levels of MHC-II, CD40, and IL-12p40 production, which were further enhanced by HFD. Acetylated-NAD had little effect on reducing the pro-inflammatory profile in BMDCs from SIRT1∆ mice, underscoring the SIRT1-NAD dependency and specially in the HFD context (Fig. 8F–I). This, coupled with lower Ido1 expression in BMDCs from SIRT1∆ mice, supports a SIRT1-IDO1 axis linked to NAD sourced from the kynurenine pathway.

Previously, we found that diminished SIRT1 levels in DCs from obese mice led to reduced accessibility at the TSS of PPARγ (Fig. 7D–F). Therefore, we examined PPARγ production in BMDCs from control (SIRT1 flox) and SIRT1∆ mice. Consistent with our ATAC-seq data (Fig. 7), PPARγ + BMDCs and mean fluorescence intensity (MFI) were lower in BMDCs from HFD-fed mice, with even further reduction in BMDCs from SIRT1∆ mice under HFD conditions (Fig. 8J, K).

In summary, our study reveals that SIRT1 is vital for regulating inflammation and metabolism in obesity. Using a Sirt1 conditional knockout mouse model, we found that the absence of SIRT1 in dendritic cells exacerbates weight gain, glucose intolerance, insulin resistance and respiratory change high-fat diet (Extended data 5). SIRT1∆ mice showed increased pro-inflammatory markers and reduced PPARγ production, connecting with the SIRT1-IDO1 axis and NAD metabolism. These findings underscore SIRT1 expressing dendric cells importance in managing obesity-related inflammation and metabolic.

## Discussion

Our study reveals that Sirtuin 1 (SIRT1) plays an important role in dendritic cell (DC) activation, phenotype, and metabolism. Furthermore, high-fat diet (HFD) reduces both the presence and the activity of SIRT1 in DCs, which culminates in greater activation of these cells, represented by the exacerbated expression of MHC Class II, costimulatory molecules, and cytokine production. In line with this, HFD directly affects the presence and activity of SIRT1, mainly through a deficit in tryptophan catabolism, which is responsible for the de novo NAD+ pathway. This coenzyme is essential for the functioning of SIRT1, which also modulates the availability of NAD+ by increasing the expression of Indoleamine 2,3-Dioxygenase 1 (IDO1), a fundamental enzyme in the catabolic synthesis of this coenzyme.

Obesity has been associated with a different pattern of DC phenotype and functionality. Previous studies demonstrated that BMDCs derived from obese animals have higher amounts of class II MHC and costimulatory molecules, which contrasts with the downregulation of IL-10, IL-4, and TGF-β production [40, 41]. These results suggest that DC progenitors in the bone marrow may emigrate to an inflammatory environment, which could potentiate the inflammatory phenotype observed in DCs from obese animals in adipose tissue. In this regard, the study by Baur and colleagues links SIRT1 to a better outcome in obesity, whereas the activation of SIRT1 by resveratrol (RES) promoted beneficial effects in HFD subjects [42]. Nevertheless, SIRT1 promotes the mobilization of fatty acids in adipocytes by improving PPAR-γ activities, thus attenuating adipogenesis in animals on a high-fat diet [43]. Additionally, previous studies have shown that SIRT1 regulates activation, cytokine production, and DC-mediated T-cell proliferation and differentiation [17, 44]. Thus, alterations in SIRT1 levels during obesity could modulate DC phenotype towards an inflammatory profile as also shown here.

Using different approaches such as SIRT1 activation by RES or inhibition by EX-527, we found that SIRT1 activity in RES-treated BMDCs/HFD decreased the expression of class II MHC, CD86, and CD40 and pro-inflammatory cytokines IL-12p40, IL-6, and TNF-α, whereas it increased the production of TGF-β. Moreover, this was associated with decreased frequency of effector Th1 cells or increased frequency of Tregs. This trend towards a tolerogenic profile in DCs mediated by SIRT1 observed by us may be related to SIRT1 activity under NF-κB since this transcription factor is an important regulator of several pro-inflammatory cytokines and surface proteins associated with the DC phenotype [45–48].

Tryptophan is an essential amino acid used in the de novo generation of NAD+, mainly from the cleavage of tryptophan itself by Indoleamine 2,3-Dioxygenase 1 (IDO1) or by isoforms 1 and 2 of Tryptophan 2,3-Dioxygenase (TDO), generating kynurenine, which can be secreted or give rise to subsequent enzymatic reactions to generate NAD+ [49, 50]. Interestingly, we observed that the supernatant from BMDCs/SD had more kynurenine and less tryptophan compared with BMDCs/HFD, and SIRT1 activation by RES increased kynurenine production by BMDCs/SD. Moreover, we found that HFD/BMDCs have impaired NAD+ production modulated by decreased kynurenine production due to less expression of IDO1. As NAD+ is an important factor to support SIRT1 function [51], our results suggest that HFD affects IDO1 regulation of the kynurenine pathway, which impairs the generation of NAD+ and consequently SIRT1 activity in DCs.

We aimed to understand how SIRT1 could be important for the regulation of the kynurenine pathway. Previous studies showed that Melanoma-derived Wnt5a promotes the transcriptional expression of IDO1 in nearby DCs by Wnt5a-β-catenin signaling and activates the PPAR-γ signaling pathway, culminating in enhanced IDO1 activity to establish an immunosuppressive microenvironment [52]. In addition, exogenous treatment of the IDO1 pathway metabolites kynurenine and quinolinic acid stimulated the formation of tumors in mice as well as the activation of β-catenin and the proliferation of human colon cancer cells [53]. Furthermore, we found a reduced accessibility at PPARγ transcription start site (TSS) in BMDCs/HFD, which aligns with our previous observations of the downregulation of Ido1 expression in BMDCs/HFD. Thus, our results suggest that HFD impairs SIRT1 activity affecting PPARγ-mediated Ido1 expression, which in turn compromises IDO1 regulation of the kynurenine pathway. These events culminate in a reduced availability of NAD+, which decreases SIRT1 activity in DCs, and it is supported by our conditional deletion of *Sirt1* in dendritic cells system (SIRT1Δ). Another factor that could be modulated in DCs in obese mice is the transcription factor FOXO3 (also spotted in our STRING analysis in Fig. 7), which is a positive regulator of SIRT1 expression. Previous studies demonstrated that mice fed with HFD have decreased expression of FOXO3 in macrophages [54]. Additionally, silencing FOXO3 in mouse DCs was also linked to decreased expression of tolerogenic mediators like indoleamine 2,3-dioxygenase (IDO1), arginase, and TGF-β, as well as increased expression of costimulatory molecules and pro-inflammatory cytokines. Thus, it is possible that downregulation of FOXO3 in obese DCs can affect the expression of SIRT1.

In summary, SIRT1 plays a central role in controlling the metabolism and phenotype of DCs. Obesity directly affects SIRT1 activity through a deficit in catabolism, which is clearly responsible for the de novo NAD+ pathway. This coenzyme is essential for the functioning of SIRT1, which also modulates the availability of NAD+ by increasing the expression of IDO1, a fundamental enzyme for this positive feedback loop.

Our findings demonstrate that the deficiency of SIRT1 in DCs affects glucose homeostasis. In line with this, glycolytic metabolism directly influences the phenotype of DCs [19, 30, 55]. and SIRT1 is a key player in cellular metabolism in different types of tissues [13, 56–58]. In this regard, our results demonstrate that SIRT1 activity in DCs regulates its metabolic profile by increasing OXPHOS and decreasing glycolytic metabolism. Thus, the alterations in the DCs phenotype in HFD may be influenced by changes in the metabolic profile of DCs mediated by SIRT1.

## Methods

### Animals

C57BL/6J animals were acquired at the vivarium of the Institute of Biomedical Sciences (ICB) of the University of São Paulo (USP), under the Ethics Committee on Animal Use (CEUA) of the ICB (no 8027280518). They were kept in SPF (specific pathogen-free) conditions with controlled temperature (25 °C), light/dark cycle (12 h/12 h), and with water and feed *ad libitum*. Each experiment performed was composed of a minimum of 3 animals and a maximum of 9 animals per group, accompanied by 2–3 experts.

To verify the presence of the desired genotype in each generation of SIRT1Δ mice (CD11c-Cre/ B6.Cg-Tg(Itgax-cre)1Reiz X Sirt1^flox/flox/ B6.129-Sirt1^tm3Fwa/J; JAX Strain #029603; JAX Strain #008068), conventional PCR was performed. We verified whether the animals possessed the CRE gene in CD11c (specifically in the promoter region of the Itgax gene) and the loxP sites in the Sirt1 gene. Reagents and protocols were followed according to The Jackson Laboratory protocols (Bar Harbor, USA). Animals positive for Itgax-cre (heterozygous) and Sirt1^flox/flox (homozygous) were considered SIRT1Δ mice, while animals positive only for Sirt1^flox/flox (homozygous) were used as control mice (SIRT1^flox).

### Glucose tolerance and insulin resistance test

For the glucose tolerance test (GTT), we injected 1 g/kg of glucose (diluted in sterile 1× PBS) intraperitoneally (Sigma-Aldrich®, Missouri, USA) after 12 h of fasting. Blood glucose concentration was measured in animals at 0, 15, 30, 60, 90, and 120 min after glucose injection, using blood glucose strips (Accu-chek®, Basel, Switzerland). For the insulin tolerance test (ITT), the animals were fasted for 8 h and received 1U/kg of regular human insulin (diluted in sterile 1× PBS) (Thermo Fisher Scientific^TM^, Massachusetts, USA) intraperitoneally. The evaluation of the glycemia of the animals at the same time intervals was carried out in the same way as for the GTT.

### High-fat diet-induced obesity model

To induce obesity, the animals (4 weeks of age) were submitted to a standard diet of 20% or high fat 60% lipids/kcal (RhosterTM, São Paulo, Brazil) with 3 g/kg of l-cystine, 2.5 g/kg of choline bitartrate, 10 g/kg of 10 g/kg of vitamin mix, 35 g/kg of G mineral mix, 100 g/kg of sucrose, 50 g/kg of microfine cellulose, 115.5 g/kg of corn starch, 132 g/kg dextrinized starch, 200 g/kg casein, 315 g/kg pork lard and 35 g/kg soybean oil for 12 weeks. After diet administration during the given period, the establishment of obesity and metabolic syndrome was assessed by monitoring weight gain, as well as by GTT and ITT tests.

### Bone marrow-derived dendritic cells (BMDCs)

To verify the presence and investigate the role of SIRT1 in DCs in vitro, bone marrow DCs isolated from the femur and tibia of lean and obese male mice were generated. After euthanasia of the animals, the femurs and tibias were collected. Muscles, cartilage and fat were removed from the bones, which were kept in 1× PBS with 2% FBS (Gibco®, Massachusetts, USA), on crushed ice (4 °C), until transferred into a laminar flow. Within the flow, both the femurs and the tibias were soaked with 70% alcohol, and, later, the ends of the bones were superficially cut with the aid of sterile scissors and forceps. After the cuts, with the aid of a BDTM syringe (California, USA) 5 mL Luer-LokTM Tip (California, USA) attached to a BDTM needle (California, USA) 0.45 × 13 mm (26 G × 1⁄2″), 2.5 mL of 1× PBS with sterile 2% FBS was injected into each bone. The injected volume was immediately filtered through a 70 μM/Nylon Cell Strainer (Corning®, New York, USA) supported under a 50 mL Falcon® tube (New York, USA) used for content. The acquired volume was centrifuged at 1500 rpm for 5 min at 4 °C. Subsequently, the supernatant was discarded and 1 ml of red blood cell lysis buffer (155 mM NH_4_Cl, 12 mM NaHCO_3_ and 0.1 mM EDTA) was added to the cell button, homogenized and incubated for 1 min at room temperature. In the next step, 5 ml of PBS with 2% sterile FBS were added to neutralize the red blood cell lysis buffer. The cells were again centrifuged at 1500 rpm for 5 min at 4 °C. The supernatant was discarded again and the cell bud was suspended with 5 ml of IMDM (Iscove’s Modified Dulbecco’s Medium - Gibco®, Massachusetts, USA) with 10% FBS and 1% Penicillin and Streptomycin (Gibco®, Massachusetts, USA). An aliquot of 2 μL of the sample was diluted 20× in Trypan Blue viability dye to count the number of cells, which occurred through the microscope and a Neubauer chamber, with the exclusion of dead cells. Finally, 1.5 × 10^6^ cells were placed per well in 6-well plates containing 2 mL IMDM with 10% FBS with 1% penicillin and streptomycin, plus 20 ng/ml GM-CSF (Recombinant Murine GM-CSF - Peprotech®, New Jersey, USA). On day 3, half the volume of the culture medium was removed, which was replaced with 1 mL of fresh IMDM.

### Pharmacological treatment of BMDCs with resveratrol, EX-527 and Acetylated-NAD

The pharmacological drugs resveratrol (catalog: R5010-100MG, Sigma-Aldrich® Missouri, USA) and EX-527 (catalog: E7034-5MG, Sigma-Aldrich® Missouri, USA) were both diluted in DMSO and stored at −20 °C, according to the manufacturer’s instructions. Dose-response tests, varying time and concentration (Appendix 1A and 1B), were performed to determine the best dose considering cell cytotoxicity and viability. After the previous tests, the concentration of 100 μM of resveratrol and the concentration of 20 μM were chosen for the other subsequent experiments, as they present greater modulation of SIRT1 expression (increase with resveratrol and inhibition with EX-527) without prejudice to viability cell. We used acetylated-NAD (2 mM) (Sigma-Aldrich®, Missouri, USA) as a direct analog of NAD to avoid complications from the metabolites of NAD precursors in our in vitro experiments. This approach also facilitates more rapid cellular absorption.

### Flow cytometry

To carry out the immunophenotyping of BMDCs and DCs identified in vivo from lean and obese animals, we used the phenotypic markers described in Table 1. The number of cells was adjusted equally between the samples of each experimental group (1 × 10^6^ cells per sample), which were acquired by BD FACSCantoTM II Cell Analyzer and BD LSR Fortessa TM Flow Cytometer (California, USA). Data were analyzed using BD FACSDivaTM and BD FlowJoXT TM software (California, USA). All the aforementioned antibodies were used in the previously standardized 1:200 μL dilution, following a previous titration performed by our laboratory.

**Table 1:** Antibodies.

fluorochrome	Target	Clone	Company
AF700	Anti-I-A/I-E	M5/114.15.2	eBioscience
Percp-cy5.5	Anti-CD80	16-10A1	BD
PE-Cy7	Anti-CD86	GL1	BD
PE	Anti-CD40	3/2.G	BD
BV421	Anti-CD11c	HL3	BD
APC-Cy7	Anti-CD11b	M1/70	BD
BV786	Anti-CD8α	53-6.7	BD
FITC	Anti-F4/80	B8	BD
FITC	Anti-CD4	H129.19	BD
PE-Cy7	Anti-CD25	3C7	Biolegend
BV711	Anti-CD45	A20	Biolegend
PE	Anti-IL12p40	JES6-5H4	Biolegend
APC	Anti-IFNγ	XMG1.2	BD
BV605	Anti-B220	C57.3	eBioscience
FITC	Anti-SIRT1	19A7AB4	Abcam
PEC-cy7	Anti-CD44	IM7	BD
AF700	Anti-CD62L	MEL-14	BD
PE	Anti-CD103	2E7	BD
NA	Anti-PPARγ	81BB	Cell Signaling
NA	Anti-β-catenin	D2U8Y	Cell Signaling
AF647	Anti-IDO1	V50-1886	BD
PE	CD64	X54-5/7.1	BD
PE	Ly-6G	1A8	Biolegend

### RNA extraction

Total RNA from cells was extracted with TRIzol TM reagent (Invitrogen TM – Texas, USA). First, 1000 μL of TRIzolTM was added to the cells, followed by a 5-min incubation at room temperature, for subsequent addition of 200 μL of chloroform, followed by a 15-s agitation and a new 3-min incubation, which was followed by centrifugation at 12,000 rpm for 15 min at 4 °C. Afterwards, the aqueous phase of the content was collected (transparent supernatant containing the RNA), which was transferred to a new 1.5 mL tube, added 500 μL of isopropanol, homogenized by vortexing and centrifuged for another 10 min at 1200 rpm and 4 °C. Subsequently, a wash was performed with 1000 μL of 75% ethanol and centrifuged for 5 min at 7500 rpm. Finally, the supernatant was discarded. After the tube was allowed to dry at room temperature for 20 min, 15 μL of water treated with the diethyl pyrocarbonate reagent (DEPEC®) was added so that the samples were quantified by the Nanodrop equipment (Thermo Fisher ScientificTM, Massachusetts, USA) and stored in −80 °C freezers until use.

### Complementary DNA (cDNA) synthesis

cDNA synthesis from 2000 ng of total RNA was performed using the M-MLV Reverse Transcriptase System Kit offered by PromegaTM (Wisconsin, USA). In a 0.2 mL tube, 2000 ng of RNA, 2.5 μL of DNAse, 5 μL of 10× reaction buffer and 1 μL of DNase-RNA free were placed in each sample for digestion of contaminating DNA. The samples were placed in the Eppendorf Mastercycler Thermocycler (Hamburgo, Germany) with a 30-min cycle at 37 °C and then incubated for 5 min on ice. After incubation, the samples were returned to the thermal cycler in a cycle of 10 min at 65 °C and a further 5 min on ice. Finally, a mixture containing 1 μL BSA (20 μg/mL), 10 μL of M-MLV Buffer 5×, 10 μL of dNTP, 2 μL of Oligo DT and 2 μL of reverse transcriptase was applied to perform the last cycle in the thermocycler. One hour at 37 °C followed by a step at 65 °C for 10 min. Samples were diluted 20× and stored at −20 °C until used.

### Real-time qPCR

Real-time qPCR (Quantitative Real Time Polymerase Chain Reaction) was performed using 5 μL of PowerUpTM SYBRTM Green Master Mix from Applied BiosystemsTM (California, USA), 2 μL of specific water for use in molecular biology from Sigma- Aldrich® (Missouri, USA), 0.5 μL of each primer (500 nM), Forward and Reverse from IDTTM (Washington, USA) and finally, 2 μL of the 20× diluted sample. All reactions were performed in 96-well MicroAmpTM Optical 96-Well Reaction Plate Strips Applied BiosystemsTM plates in the QuantStudio 12 K Flex Real-Time PCR System Applied BiosystemsTM instrument. The sequences of the primers of the analyzed genes were: Sirt1 F: 5′-AAACAGTGAGAAAATGCTGG-3′, R: 5′-GGTATTGATTACCCTCAAGC-3′; pro-Il-1b F: 5′-TGGACCTTCCAGGATGAGGACA-3′, R: 5′-GTTCATCTCGGAGCCTGTAGTG-3′;Il6 F: 5′-TACCACTTCACAAGTCGGAGGC-3′, R: 5′-CTGCAAGTGCATCATCGTTGTTC-3′; Il12b F: 5′-GGCCATGAGGCTGGATCTC-3′, R: 5′-AACATTTGAATCCTGCAGCCA-3′; Il12a F: 5′-TTTTCTGGCATCTCCCCCTGTG-3′, 5′-TGGGTGGGTCAGGTTTGATGATG-3′; Ifna F: 5′-GAAATTCCTGATCCAGACAAAAAC-3′, R: 5′-ATCACTTCAATGGCCTCTTGTGTAG-3′; Ifnb F: 5′-CAACTTGCTTGGATTCCTACAAAG-3′, R: 5′-TATTCAAGCCCTCCCATTCAATTG-3′; Tnfa F: 5′-TGTGCCCCGTATCCAGTGT-3′, R: 5′-CGGATCCTTTGCAAGCAGAA-3′, Il10 5′-ATTTGAATTCCCTGGGTGAGAAG-3′, R: 5′-CACAGGGGAGAAATCGATGACA-3′ Tgfb1 F: 5′-GCAGCACGTGGAGCTGTA-3′; Il35 (Ebi3) F: 5′-GTTCTCCCACGGTGCCCTA-3′, R: 5′-CGGCTTGATGATTCGCTC-3′; Ido1 5′-AGGATCCTTGAAGACCACCA-3′, R: 5′-CCAATAGAGAGACGAGGAAG-3′; Bactin F: 5′-TGAGAGGGAAATCGTGCGTG-3′, R: 5′-TGCTTGCTGATCCACATCTGC-3′. All primer sequences were purchased from the company IDTTM at a concentration of 100 nM and diluted in a standard way to 10X for use, which were previously titrated, reaching the standard of 0.5 μL in 10 μL of final reaction, with the concentration 500 nM of each primer in the pair.

### Co-culture between ova pulsed BMDCs and OT-II animal-derived naive CD4 T cells

Initially, BMDCs were generated as previously described, and on day 6 the cells were treated with Resveratrol (100 μM) or EX-527 (20 μM) and with Ovalbumin (OVA) Sigma-Aldrich® (Missouri, USA) at the concentration of 0.1 mg/mL previously filtered through a 0.45 μm filter in 1× PBS. On day 7, BMDCs were removed from the 6-well plate, isolated by EasySepTM Mouse CD11c Positive Selection Kit II Stem CellTM (Los Angeles, USA) and replaced in a 96-well U-bottom plate, with 25 × 10^4^cells per well. With the naive CD4+ T lymphocytes (CD4+62DLhigh) properly isolated from the lymph nodes and spleen of OT-II animals by the EasySepTM Mouse Naïve CD4+ T Cell Isolation Kit from Stem CellTM (Los Angeles, USA), CellTraceTM Violet Cell was added Proliferation from Thermo Fisher ScientificTM (Massachusetts, USA) at a concentration of 5 μM. The cells were subsequently incubated at 37 °C, 5% CO_2_, for 20 min in 1 mL of complete RPMI 1640 medium, that is, containing 5% FBS, 1% penicillin, streptomycin, 1% l-glutamine, 1% sodium pyruvate, 1% non-essential amino acids, 25 mM HEPES and 1:1000 dilution of 2-β-Mercaptoethanol (Gibco® of Massachusetts, USA). After that, it was added to the 96-well plate already containing the 25 × 10^4^ BMDCs, 1 × 10^5^ CD4+ T lymphocytes, naive for 3 days. On day 8, the wells containing both cells were submitted to the addition of 50 ng/mL of PMA, 1 μg/mL of ionomycin and 1 μg/mL of brefeldin A BDTM solution (California, USA) and subsequently incubated for 5 h in 37 °C at 5% CO_2_. CD4+ T lymphocyte proliferation and cytokine production were analyzed by flow cytometry using the BD FACSCanto™ II Cell Analyzer and BD LSRFortessa™ Flow Cytometer (California, USA). Data were analyzed using BD FACSDivaTM and BD FlowJoXT TM software (California, USA).

### Liquid chromatography coupled to mass spectrometry (LC–MS)

The quantification of tryptophan metabolites was performed by liquid chromatography coupled to triple quadrupole LC-MS/MS (QqQ) mass spectrometry. The equipment used is composed of LC Pumps 1250 Bin Pump VL, auto-injector 1260 HiP ALS coupled to a 6460-mass spectrometer, all from Agilent Technologies TM (Massachusetts, USA). Analyzes were performed in Mass HunterTM software and data collected in MRM (Multiple Reaction Monitoring) mode. For sample preparation, the culture supernatant (500 μL) was transferred to a 1.5 mL microtube containing 1 mL of methanol: acetone (1:1) with 0.1% acetic acid. After adding the samples, 10 μL of a mix of internal standards (MLT-D4 and Trp-D5) for the analytes studied was added to the microtube. Subsequently, we homogenized by vortexing for 1 min and then stored these solutions for half an hour at −20 °C for precipitation of proteins from the culture medium. After incubation, the microtubes were subjected to centrifugation at 14,000 × *g* for 10 min at 4 °C. The supernatant from the microtubes was transferred to new microtubes and dried in Speedvac®. At the end of drying, the samples were reconstituted in 100 μL of a mixture of water:methanol (9:1) and then analyzed by liquid chromatography.

### Metabolic analysis

One day prior to the experiment, 200 μL of Agilent Seahorse XF Calibrant (Massachusetts, USA) was added to each well of the cartridge (Massachusetts, USA), which was hydrated and incubated for 24 h at 37 °C without CO2.

Oxygen consumption rates (OCR) were quantified on an XF-96 extracellular flow analyzer (XF-96 Extracellular Flux Analyzer - Seahorse®, Bioscience) to obtain indications of mitochondrial respiration. For this, BMDCs positively selected by the EasySepTM Mouse CD11c Positive Selection Kit II Stem CellTM column (Los Angeles, USA), from lean and obese animals, with and without Resveratrol (100 μM/24 h) or EX-527 (20 μM/24 h), were plated on a specific plate for XF-96 Extracellular Flux Analyzer assays, which had 96 wells with 50,000 cells/well containing 200 μL of Agilent Seahorse XF RPMI Medium (Massachusetts, USA), supplemented with 1 mM of l-glutamine, Pyruvate and 10 mM glucose (Gibco®, Massachusetts, USA). Subsequently, after calibration of the XF-96 Extracellular Flux Analyzer equipment, oligomycin drugs were injected at 1 μM, 5 µM CCCP (Carboxyl-carbonyl cyanide phenylhydrazone); and antimycin-A/rotenone to 1 μM. In this way, it was possible to obtain, in real-time, the basal respiration, protons, reserve capacity, oxidative phosphorylation, and maximal respiratory capacity of the cells under study.

BMDCs with the same pharmacological treatments for SIRT1, which were maintained in 200 μL/well of Agilent Seahorse XF RPMI Medium (Massachusetts, USA) supplemented with 1mM l-glutamine and 10 mM glucose (Gibco®, Massachusetts, USA), passed through the XF-96 Extracellular Flux Analyzer to measure the extracellular acidification rate (ECAR) with the following drugs: glucose (100 mM), oligomycin (1 μM) and 2-DG (50 mM). Thus, the indices of glycolysis, glycolytic capacity and glycolytic reserve were calculated. Values were standardized according to the number of cells for both OCR and ECAR experiments. In addition, all outliers were removed by the specific test provided by the GraphPad Prism 6®/8® software.

### SIRT1 enzymatic activity

SIRT1 activity was determined with a SIRT1 fluorometric kit (Enzo® Life SciencesTM, New York) according to the manufacturer’s instructions. Briefly, this assay uses a small lysine-acetylated peptide, corresponding to human p53 K382. The lysine residue is deacetylated by SIRT1, and this process is dependent on the addition of exogenous NAD+. This exogenous NAD+ was necessary, probably because endogenous NAD+ was lost during sample preparation. SIRT1 inhibitors, nicotinamide (2 mM), suramin (100 μM) and sirtinol (100 μM) were used to confirm the specificity of the reaction. The samples were homogenized in the NETN buffer provided by the kit. The homogenates were then incubated for 10 min at 37 °C to allow degradation of any NAD+ contaminants. Next, 10 mM DTT was added to the medium and the homogenates were incubated again for 10 min at 37 °C. The 25 μg protein/well homogenates were then incubated in SIRT1 assay buffer in the presence of Fluor Lys substrate – SIRT1 at 100 μM (Enzo® Life SciencesTM) and 5 μM TSA® to determine activity independent of SIRT1 (control) or with the same substrate. About 5 μM TSA and 200 μM NAD+ were used to determine SIRT1-dependent activity. After 1 h of incubation at 37 °C, the reaction was terminated by the addition of a solution containing Fluor de Lys Developer (Enzo® Life SciencesTM) and 2 mM nicotinamide. The plates were incubated at 37 °C for 1 h. Values were determined on a fluorometric plate reader (Synergy HTX® from Biotek) with an excitation wavelength of 360 nm and an emission wavelength of 460 nm. The fluorescence calculation included the subtraction of a blank consisting of a buffer without NAD +, which was expressed as a percentage relative to the control (dotted line in the graph). In all cases, we confirmed the linearity of the reaction over time.

### Confocal microscopy

The BMDCs previously isolated by column (as was done in the proliferation assay) were plated in 12-well plates containing Round Glasses Coverslips® (VWRInternationalLLCTM, Pennsylvania, USA). After adherence, the cells were fixed with 4% PFA for 15 min at room temperature. environment. The PFA was aspirated and two washes (5 min each) were done with ice-cold 1× PBS. Then, the samples were permeabilized with a solution consisting of 1× PBS, 0.2% triton x-100 and 1% BSA for 2 h at 4 °C. Again, after aspirating the permeabilization solution, coverslips were incubated overnight with the SIRT1 primary antibody (D1D7, Cell Signaling Technology®, Massachusetts, USA) at a 1:500 dilution in permeabilization solution. The following morning, coverslips were washed 3× with ice-cold 1× PBS, for subsequent incubation with anti-rabbit AF555 secondary antibody (Abcam® concentration and clone, Cambridge, UK) for 1 h at 37 °C in the dark. After another 3 washes with ice-cold 1× PBS, coverslips were incubated at 10 μg/mL with Hoechst® (H3570, Thermo Fisher ScientificTM, Massachusetts, USA) and Phalloidin Dylight AF488 (21833, Thermo Fisher ScientificTM, Massachusetts, USA). After the last wash with ice-cold 1× PBS, slides were mounted with CS7703/DAKO mounting medium (Agilent, Massachusetts, USA). Images were acquired by Multiphoton confocal, Zeiss LSM-780 NLO (ZeissTM, Oberkochen, Germany) and analyzed by ImageJ®.

### Database bioinformatic analysis

Through the transcriptome data made available by the publication of GSE42432, published on the GEO2R platform (https://www.ncbi.nlm.nih.gov/geo/geo2r/), the samples were separated into two groups, as previously done (Konings et al., 2014): 9 patients treated with placebo (Mock) and 9 patients treated with RES. Subsequently, data normalization was verified by the GEO2R platform itself, which in turn generated the list of altered genes evaluated by RNAseq in Log in base 2, also identified as mRNA intensity. The same was performed on the GSE59034 data set regarding *sirt1* and *ido1* mRNA levels. SIRT1 and IDO1 values were extracted from the database, plotted and evaluated by the analysis of the t-student test, using the GraphPad Prism 8® software. In addition, these same genes, considered differentially expressed between the groups analyzed, were submitted to an enrichment analysis by the Metascape website algorithm [55], which in turn, generated the metabolic pathways (via KEGG pathway analysis) and cell signaling (via GO biological analysis) in the order of –Log10(P).

### ATAC-seq

Fifty thousand BMDC/SD or /HFD cells were submitted to OMNI-ATAC-seq [59]. Briefly, cells were centrifuged at 500 rcf for 5 min at 4 °C and resuspended in lysis buffer (10 mM Tris-HCl, pH 7.4, 10 mM NaCl, 3 mM MgCl_2_, and 0.1% Tween-20). Tagmentation was performed using 2.5 μl Tn5 transposase in TM buffer (20 mM Tris-HCl pH 7.6, 10 mM MgCl_2_, 20% Dimethyl Formamide, 0.1% Tween-20, 0.01% Digitonin, 1× PBS) and incubating at 37 °C for 40 min. Library construction was using the Nextera DNA Library Prep Kit (Illumina, 15028212). Quality control of prepared libraries was conducted using Agilent 2100 Bioanalyzer for fragment analysis. Libraries were pooled to equimolar concentrations using the KAPA Library Quant Kit (cat# KK4854) and sequenced with paired-end 75 bp reads on an Illumina HiSeq2500 instrument.

### ATAC-seq data analysis and code availability

Paired-end sequencing reads were trimmed using CutAdapt [60] and aligned to the reference mouse mm10 assembly using Bowtie 2 [61]. Picard MarkDuplicates tool was used to mark duplicate reads and BAM files were filtered with SAMtools [62] to discard unmapped reads, those which were not the primary alignment, reads failing platform/vendor quality checks, and PCR/optical duplicates (-f 2 -F 1804). Quality analysis was assessed with ataqv [63]. Peak calling was performed using MACS2 with -q 0.01 and –SPMR parameters and visualized with IGV2.11 software. BEDTools-2.29 [64] was used to intersect significant peaks between replicates. DiffBind [65] was used to plot PCA, contrast SD and HFD samples, perform differential accessibility analysis with DESeq2 function, and plot heatmaps with plot peak profile function. Genes with overlapping peaks at TSS were identified with ChIPseeker [66]. GO Functional enrichment was performed using clusterProfileR [67] and volcano plot of DA genes was generated using Enhanced Volcano [68]. Code availability on https://github.com/goescp/limaolsen.

### Total NAD/NADH colorimetric assay

Bone marrow-derived dendritic cells (BMDCs) were prepared by seeding them into 96-well plates at a density that would allow the cells to achieve the desired confluence within 24 h. The plates were incubated under standard cell culture conditions, typically at 37 °C with 5% CO_2_.For the assay, samples were prepared following the manufacturer’s instructions precisely (Promega, catalog G9071). Following reagent preparation, the assay was conducted by adding the reagents to the wells containing the BMDCs. The incubation conditions (e.g., temperature and duration) were as specified in the protocol to ensure optimal reaction development. To measure the NAD+ levels, a plate reader was used according to the manufacturer’s recommendations. The reader was calibrated to the appropriate wavelength for detecting luminescence. For data analysis, a standard curve or calibration data provided with the assay kit was employed to quantify NAD+ levels in the samples. The NAD+ concentrations were compared across different treatment conditions to assess the effects of experimental variables. Positive and negative controls were included to validate the accuracy and reliability of the results.

The quantification of NAD+ was verified using the standard curve to ensure accurate measurement. Data interpretation involved analyzing the intensity of the assay signal, with higher intensities indicative of higher NAD+ levels. This data was then compared to NADH levels, and the NAD+/NADH ratio was calculated and plotted in the graphs for further evaluation.

### Statistical analysis

When determining the sample size for research involving 3R (Replacement, Reduction, Refinement) mouse use, we base our decision on a combination of ethical considerations and statistical evidence. Ethically, the principle of Reduction emphasizes minimizing the number of animals used while still obtaining valid results. Statistically, appropriate sample size calculation is crucial to ensure the study is adequately powered to detect meaningful effects and avoid Type I and Type II errors. Also, we consider the variance with each group, which were not highly significant beyond the regular biological variance within individuals.

Differences between groups were assessed by student t-test analysis, ANOVA or AUC (Area Under the Curve) when appropriate. The results were presented as mean ± standard deviation for parametric and non-parametric variables previously submitted to the normality test. All analyses were calculated using the GraphPad Prism 6®/8® software and differences will be considered significant when the adjusted *p* value is less than or equal to 0.05.

## Supplementary information

Supplementary Figures and legends

## Data Availability

The ATAC-seq data supporting the findings of this study are available on GitHub at https://github.com/goescp/limaolsen. Additional data or specific requests can be made by contacting the corresponding author. Data will be made available upon reasonable request, subject to appropriate ethical considerations.
