# Supplementary material for: Sirtuin 1 regulates the phenotype and functions of dendritic cells through Ido1 pathway in obesity
Source: Cell Death Dis. 2024 Oct 18;15(10):757. doi: 10.1038/s41419-024-07125-3 (PMC11489582; doi:10.1038/s41419-024-07125-3)
Supplement: Supplementary file 1 — Supplementary Figures and legends [file 41419_2024_7125_MOESM1_ESM.pdf]

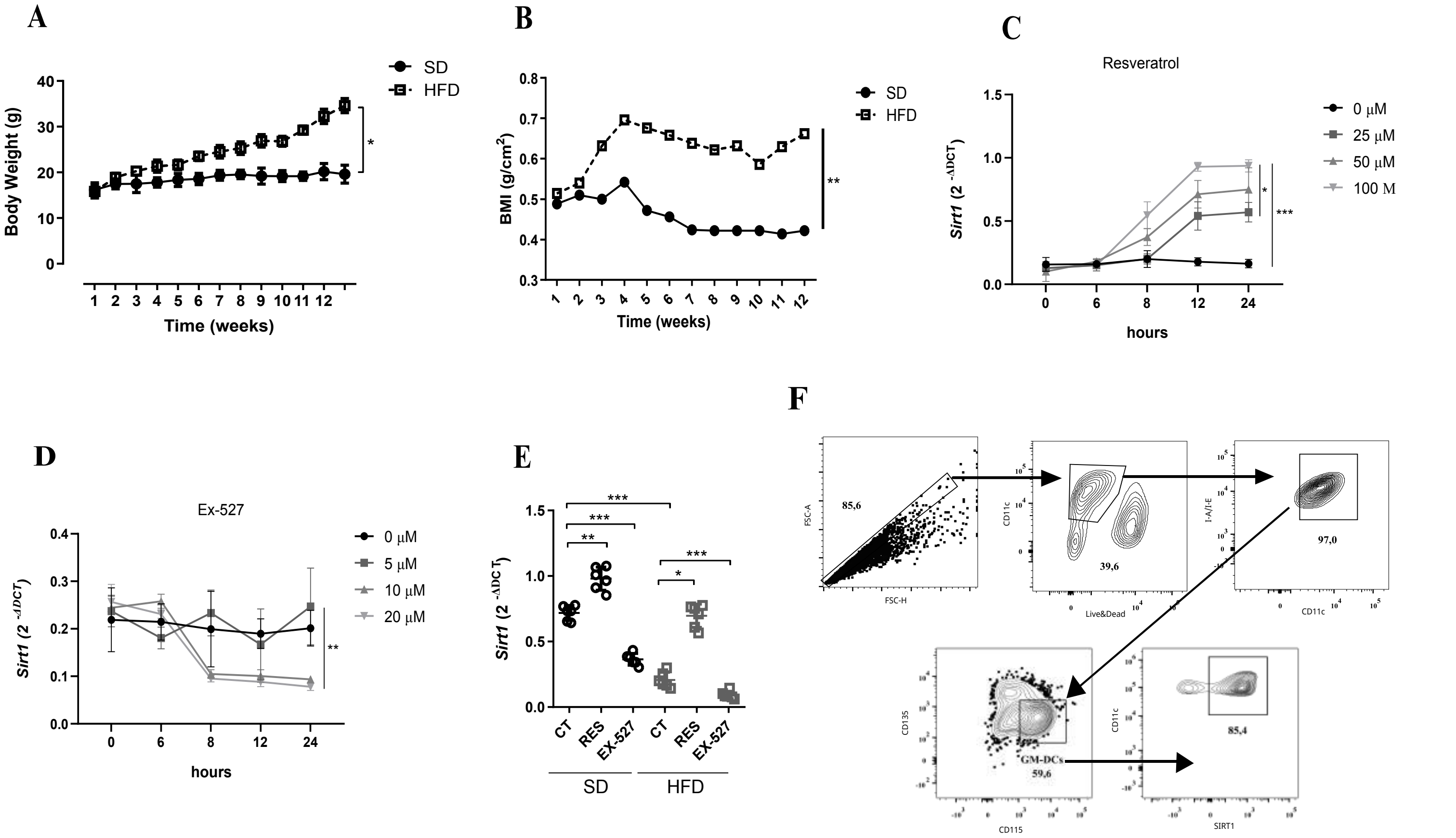

Extended data 1. Weekly weight gain for C57B6/j mice (8-10 weeks) on standard diet (SD) and high-fat diet (HFD) (A). Body Mass Index (BMI [g/cm²]) after 12 weeks of standard diet(SD) or high-fat diet (HFD) (B). Resveratrol titration through qPCR for Sirt1 expression in BMDCs(C). EX-527 titration with qPCR for Sirt1 expression in BMDCs (D). qPCR of Sirt1 from BMDCs ofSD mice and HFD mice, which were treated with Resveratrol (50 μM), EX-527 (10 μM), or withthe vehicle (CT [DMSO]) (E). Gate strategy from flow cytometry data to determine GM-BMDCs,Singlets+Live Dead-CD11c+MHCII+CD115+ (F). Also, to observe the percentage of GM-BMDCswhich had SIRT1 (flow cytometry data). The significance values (p) are indicated as follows: * =p <0.05; ** = p<0.01; *** = p<0.001; **** = p<0.0001, determined by One-way ANOVA or t-testwhen necessary, using GraphPad Prism®. The graphs and illustrations represent arepresentative experiment of three distinct experiments, with 3-6 animals per group. experiments.

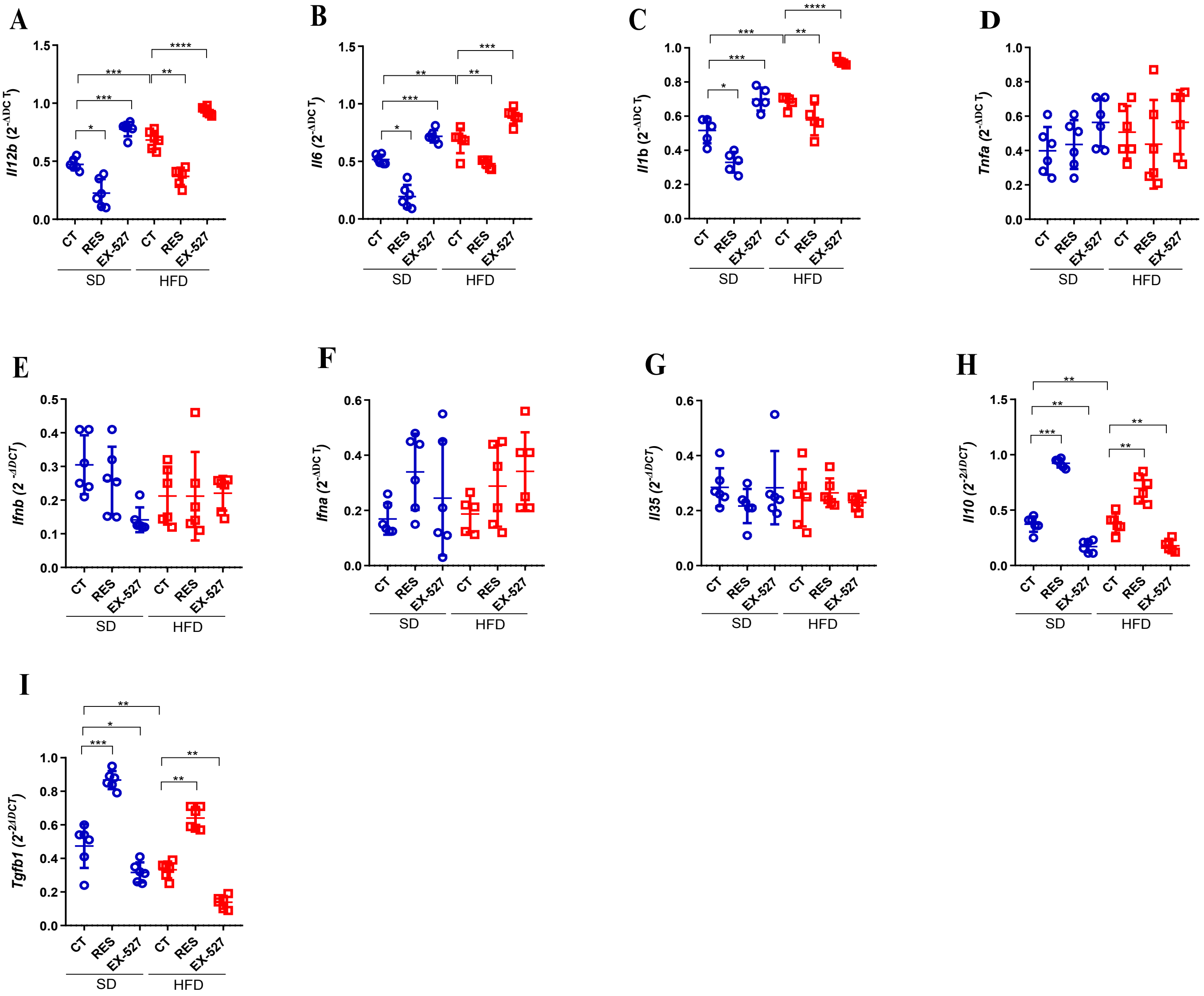

Extended data 2. Representing qPCR data (2-ΔΔCT) of the main cytokines produced byBMDCs/SD and BMDCs/HFD under RES [50 μM], EX-527 [10 μM] treatment, or no treatment (CT).Cytokines include IL-12b (A), IL-6 (B), pro-IL-1b (C), TNFα (D), IFNβ (E), IFNα (F), IL-35 (G), IL-10 (H), and TGFβ (I). The significance values (p) are indicated as follows: * = p <0.05; ** = p <0.01;*** = p <0.001; **** = p <0.0001, determined by One-way ANOVA or t-test when necessary, usingGraphPad Prism®. The graphs and illustrations represent a representative experiment of twodistinct experiments, with 3-6 animals per group.

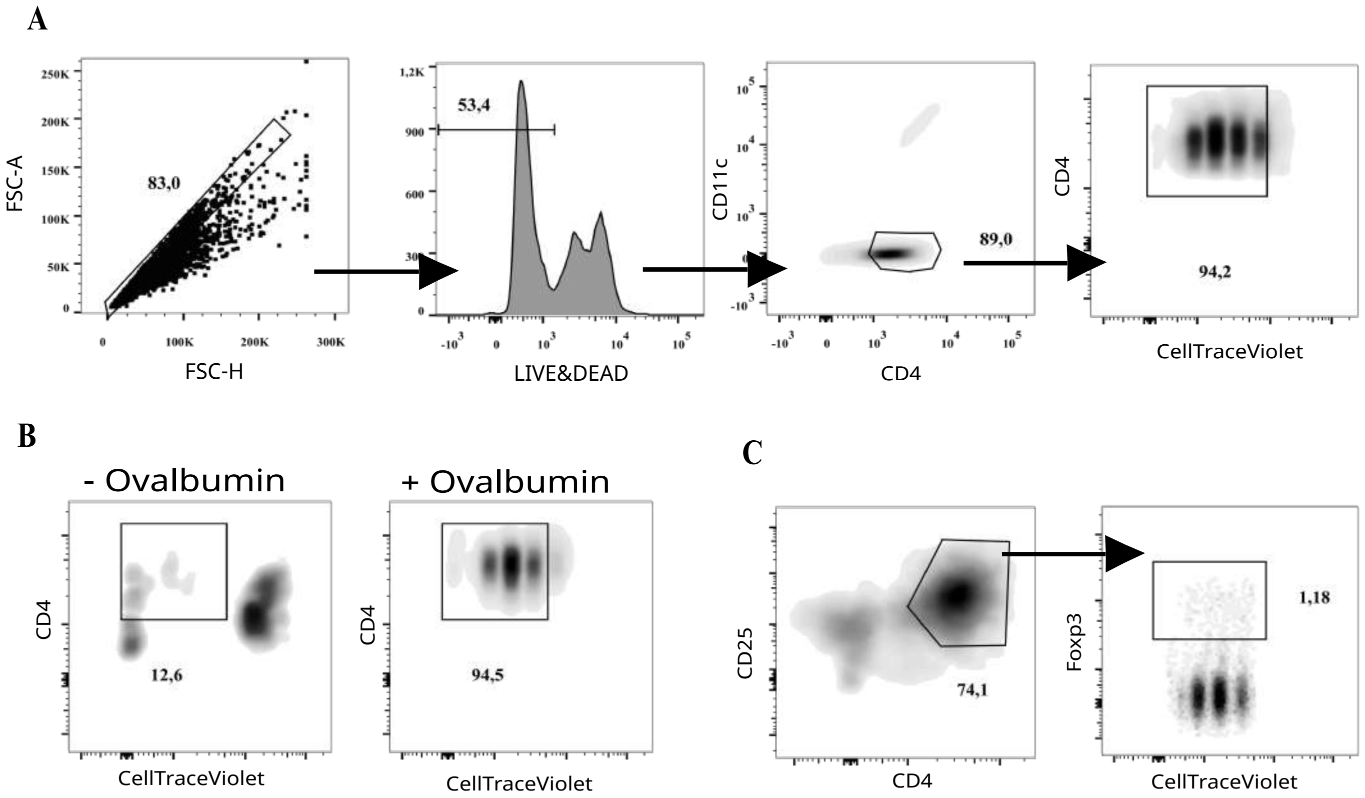

Extended data 3. Gate strategy for OT-II CD4 T cells co-cultured with BMDCs/SD andBMDCs/HFD treated (RES or EX-527) or not treated (A). OT-II CD4 T cells co-cultured with BMDCs pulsed (+) and without (-) Ovalbumin (B). Gate strategy for Treg (CD4+CD25+Foxp3+)(C). The significance values (p) followed the order: * = p <0.05; ** = p <0.01; *** = p <0.001; ****= p <0.0001, which were determined by the One-way ANOVA test or t-test when necessary, usingGraphPad Prism®. The graphs and illustrations represent a representative experiment of threedistinct experiments, with 3-6 animals per group..

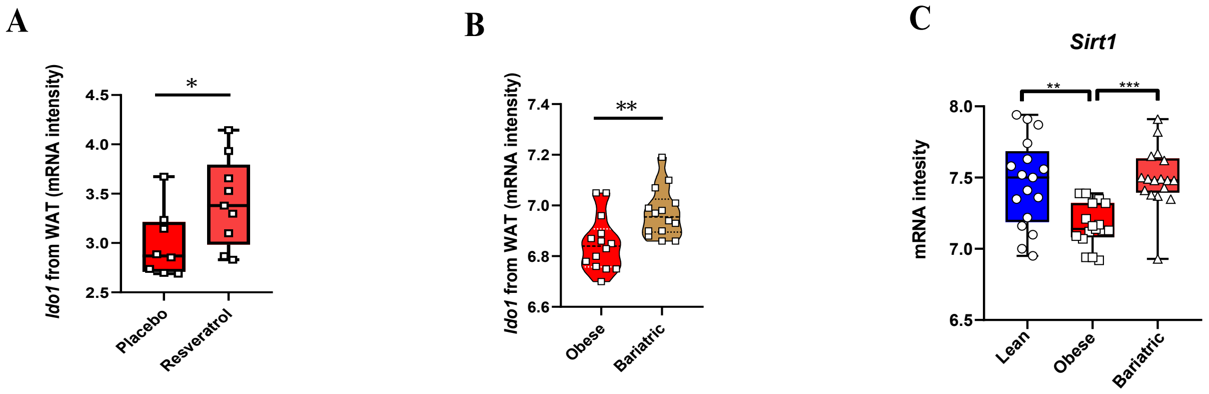

Extended data 4. Analysis by Geo2R database platform GSE42432, which represents theintensity of mRNA (Log2) of IDO1 from white adipose tissue RNAseq data from patients treatedwith placebo and 150 mg/ml of resveratrol (RES) (A). Ido1 mRNA intensity (Log2) of RNAseqdataset (GSE59034) from human white adipose tissue before (obese) and after (bariatric)bariatric surgery (B). Additional lean control was also included to compare SIRT1 expressionlevels from GSE59034 (C). The significance values (p) followed the order: * = p <0.05; ** = p <0.01;*** = p <0.001; **** = p <0.0001, which were determined by the One-way ANOVA test or t-testwhen necessary, using GraphPad Prism®. The graphs and illustrations represent arepresentative experiment of three distinct experiments, with 6-12 patients per group.

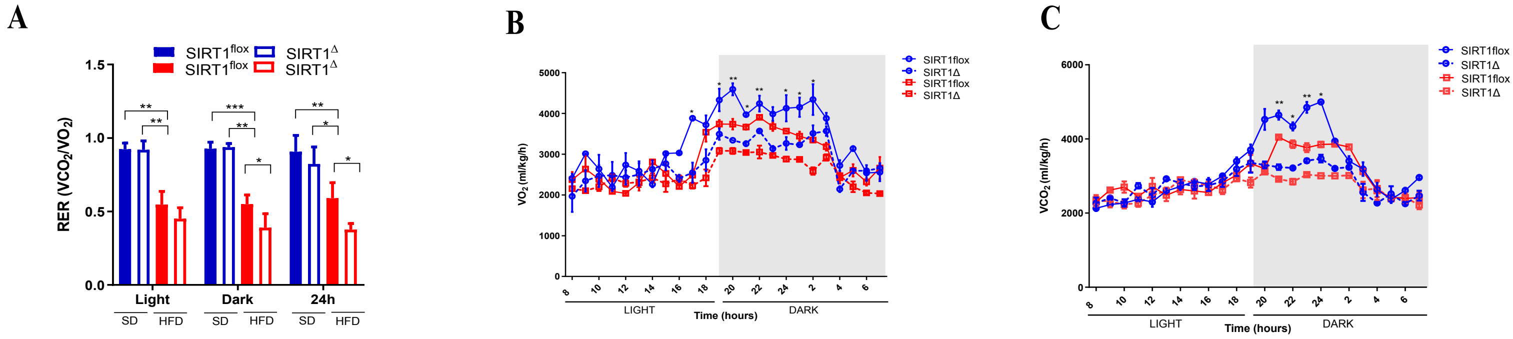

Extended data 5. Respiratory Exchange Ratio (RER) - VCO2 produced divided by VO2consumed (where V is volume) by SIRT1flox and SIRT1Δ mice under standard diet (SD – blue) and high-fat diet (HFD – red) (A). VCO2 (B) VO2 (C) consumed by SIRT1flox and SIRT1Δ miceunder SD and HFD. The significance values (p) followed the order: * = p <0.05; ** = p <0.01; *** =p <0.001; **** = p <0.0001, which were determined by the One-way ANOVA test or t-test whennecessary, using GraphPad Prism®. The graphs and illustrations represent a representativeexperiment of two distinct experiments, with 4-6 mice per group.
